# Current status of nurses’ public health emergency response capacity for emerging infectious disease: a cross-sectional study

**DOI:** 10.3389/fpubh.2025.1612790

**Published:** 2025-08-06

**Authors:** Qin Qiu, Yuntong Liu, Fanping Meng, Xinhua Wang, Chunxiang Su

**Affiliations:** ^1^School of Nursing, Beijing University of Chinese Medicine, Beijing, China; ^2^Fifth Medical Center of the PLA General Hospital, Beijing, China; ^3^302 Clinical Medical College, Peking University, Beijing, China

**Keywords:** current status, nurse, public health, emergency response capacity, emerging infectious disease

## Abstract

**Objectives:**

This study aimed to assess the current status of nurses’ public health emergency response capacity for emerging infectious diseases (EIDs) in tertiary hospitals in Beijing, explore the deficiencies of nurses’ emergency response capacity, and analyze the influencing factors in the post-epidemic era.

**Methods:**

A convenience sampling method was utilized to recruit registered nurses in 3 tertiary hospitals in Beijing from 27 August and 2 September 2024. The research team designed a questionnaire that included basic information section and a section of questionnaire on public health emergency response capacity for EIDs. The overall Cronbach’s *α* of the questionnaire was 0.982. The non-parametric Mann–Whitney U test and Kruskal-Wallis test were employed to assess intergroup differences, subsequently leading to the development of a multiple linear regression model based on the study data.

**Results:**

The study enrolled 1,484 registered nurses. A total of 1,446 valid questionnaires were maintained, resulting in a 97.4% valid response rate. The median score of nurses’ public health emergency response capacity for EIDs was 177, indicating a modest level of competency. The analysis revealed statistically significant disparities in the scores of the dimensions of nurses’ emergency response competencies (H = 1146.228, *p* = 0.000). The scores of the dimensions were ranked in ascending order as follows: cognition (median = 3.00, IQR = 2.50–3.20), theoretical knowledge (median = 3.00, IQR = 2.75–3.46), disposal capacity (median = 3.36, IQR = 2.93–4.00), and clinical skill (median = 3.86, IQR = 3.29–4.36). There was a significant difference in scores for nurses’ public health emergency response capacity for EIDs in terms of clinical role (*p* = 0.008), hospital classification (*p* = 0.000), and rescue experience (*p* = 0.000). Multiple regression analysis revealed that the hospital classification and rescue experience of EIDs were the influential factors of nurses’ emergency response capacity (R^2^ = 0.044, *F* = 11.023, *p* < 0.05), and rescue experience significantly predicted emergency response capacity scores.

**Conclusion:**

The findings revealed that the nurses’ public health emergency response capacity for EIDs was at a moderate level and the capacity of the dimensions were unbalanced, necessitating targeted improvement. Future initiatives should prioritize strengthening theoretical reinforcement training and cognitive transformation programs for nurses, while accelerating the development of EID specialty nurses and inter-hospital collaboration. Existing training and educational mechanisms require optimization, with particular emphasis on organizational incentives alongside leadership modeling. Ensuring that nurses will have better performance in future public health emergencies for EIDs.

## 1 Introduction

Public health emergencies refer to sudden event that causes or may cause serious harm to human health, including severe infectious diseases, diseases occurring in clusters with ambiguous etiologies, severe food or occupational poisoning, and other incidents that significantly impact public health (1). Emerging infectious disease (EID) is a significant public health emergency, representing roughly 87.5% of all reported public health emergencies (2). In recent years, EIDs have occurred with increasing frequency (3). The World Health Organization (WHO) reported that 168 countries experienced over 1,200 infectious disease outbreaks between 2012 and 2017 alone, including severe acute respiratory syndrome, dengue fever, Ebola virus disease, and H1N1 influenza, etc. (4). Especially, the pandemic of coronavirus disease 2019 has posed unprecedented challenges to global health security and healthcare infrastructures worldwide (3, 5, 6). This crisis has shown significant shortcomings in public health emergency response capabilities in the EIDs (7).

The WHO Strategic Framework for Emergency Preparedness clearly asserts that building health system resilience is fundamental to emergency preparedness (8). Its core is the establishment of a health workforce which is adequately staffed and which has an appropriate and equitably distributed mix of skills and competencies. Besides, this workforce must be properly remunerated, supported and motivated to carry out its duties in routine and emergency circumstances. Within this framework, hospitals serve as the primary institutions for medical intervention, and nurses as the core workforce for emergency response in EIDs (7, 9, 10). The competence of nurses to effectively implement medical emergency response measures during public health emergencies (hereinafter termed emergency response capability) (11) is of great significance to ensure the successful completion of the rescue missions (12–14). This capability directly influences the management efficacy of public health emergencies in a region or a country (15). Therefore, it is necessary to study the nurses’ emergency response capacity for EIDs.

However, current evidence (16, 17) have shown that nurses’ public health emergency response capacity for EIDs is suboptimal and requires enhancement (10, 18, 19). Moreover, the criteria for nurses’ emergency response competence vary over time (20). Evidence demonstrated that nursing competency levels significantly predict outbreak containment success rates. Inadequate knowledge of emergency response and unstandardized emergency procedures increased adverse outcomes for both nurses and patients (11, 21). Therefore, examining nurses’ public health emergency response capacity for EIDs is necessary to improve the overall quality of rescue, medical care, and the health and safety of nursing staff. Furthermore, although the WHO framework emphasizes the necessity for continuous capacity development of the health workforce, research on the mechanisms influencing nurses’ emergency response capabilities in the post-pandemic era remains are still little explored. Therefore, guided by the WHO Strategic Framework for Emergency Preparedness, we focused specifically on the nursing workforce domain delineated within the framework. The study sought to assess the current situation of nurses’ emergency response capacity at tertiary hospitals in Beijing, explore the weaknesses of nurses’ emergency response capacity, and analyze the factors in the post-epidemic era. Lastly, we hope that it can provide an evidence-based foundation for improving the emergency response ability of nurses, formulating targeted training programs and creating a comprehensive evaluation system aligned with the WHO Strategic Framework.

## 2 Materials and methods

### 2.1 Study design

This cross-sectional study utilized a convenience sampling method to recruit registered nurses from 3 tertiary hospitals in Beijing between 27 August and 2 September 2024. All procedures were conducted following the Declaration of Helsinki (22). The article was prepared according to the standards for reporting observational studies of the Strengthening the Reporting of Observational Studies in Epidemiology (STROBE) (23).

### 2.2 Ethical considerations

This study was permitted by the Ethics Committee of the Fifth Medical Center of the PLA General Hospital (KY-2024-8-124-1).

### 2.3 Participants

Inclusion criteria: Registered nurses in Beijing’s tertiary hospitals.

Exclusion criteria: (1) registered nurses who did not want to participate in this survey; (2) nurses who withdrew from the study for any reason during the investigation period.

### 2.4 Tools

The questionnaire was designed by the research team and consisted of two parts: sociodemographic characteristics of participants and a validated emergency response capacity assessment scale for EIDs.

#### 2.4.1 Sociodemographic characteristics

It included gender, age, and educational background. Work-related information included professional rank, clinical role, department, clinical experience, hospital classification, and rescue experience.

#### 2.4.2 The questionnaire on nurses’ public health emergency response capacity for EIDs

The questionnaire was developed based on the WHO Strategic Framework for Emergency Preparedness has four dimensions: cognition for EIDs (10 items), theoretical knowledge for EIDs (16 items), clinical skill for EIDs (14 items), and disposal capacity for EIDs (14 items). The questionnaire is a 5-point Likert scale, with responses ranging from 1 (not at all) to 5 (very familiar), yielding a total score range of 54–270. A higher overall score indicates superior emergency response capacity. Scores below 60% indicate poor emergency response capacity, scores ranging from 60–79% signify moderate emergency response capacity, scores between 80–89% denote good emergency response capacity, and scores ≥ 90 90% reflect excellent emergency response capacity. The overall Cronbach’s *α* of the questionnaire was 0.982. The Kaiser-Meyer-Olkin (KMO) test coefficient for the questionnaire was 0.979. The questionnaire demonstrated strong validity and reliability.

### 2.5 Data collection

This research was a cross-sectional survey done by an anonymous online questionnaire from 27 August to 2 September 2024, at three tertiary hospitals in Beijing. Methodological literature indicates that the sample size should be 10–15 times the number of items (11), taking into account rejection and questionnaire invalidity of no more than 20%. The total number of variables in this study was 62 and the required sample size was 775 to 1,163. The researchers sent a questionnaire link to the head nurse of each clinical unit via WeChat to recruit participants. The questionnaire was designed with uniform instructions for completion. The participants were informed of the study protocol and e-consent for the study online. Participants read the consent form before the start of the survey and checked a box indicating that they understood and agreed with the content presented in the survey. Anonymity was maintained during data collection. Participants were allowed to withdraw from attendance at any time without negative consequences. All data were handled with confidentially. The head of each nursing unit could encourage but should not force nurses to participate in this research. To ensure the validity of the questionnaires and reduce research bias, participants were asked to fill out the questionnaire once with a unique IP address. Additionally, after data collection, questionnaires completed in under two minutes were excluded based on findings from the pilot survey.

### 2.6 Data analysis

The data were checked by two researchers and then imported into IBM Statistics Package for Social Sciences (SPSS) Version 20.0 for statistical analysis. Descriptive statistics such as frequencies, percentages, means, medians, and standard deviations were used to describe and summarize variables. If the data exhibited non-normal distribution patterns (Kolmogorov–Smirnov test *p* < 0.05), the non-parametric Mann–Whitney U test and Kruskal-Wallis test were used to test statistically significant differences between the groups. Multivariable linear regression models were constructed, using the nurse’s public health emergency response capacity for EIDs and its corresponding dimensional capacities as the dependent variables and demographic variables as independent variables. The level of significance was set at *p* = 0.05.

## 3 Results

A total of 1,484 questionnaires were received in this study. After excluding invalid questionnaires with incomplete data, 1,446 valid questionnaires were retained with 97.4% valid response rate. Among the 1,446 participating nurses, 96.1% were female and 3.9% were male. These nurses come from different departments, including operating theatres, emergency departments, internal medicine, surgical, and intensive care units. The age of the nurses ranged from 18 to 57 (median = 30, IQR = 26–35) years. The details of the participants are shown in Table 1.

**Table 1:** General information of the participants (*n* = 1,446).

Variable		*N* (%)/median (IQR)
Age (years)		30.00 (26.00–35.00)
Gender	Female	1,389 (96.10)
	Male	57 (3.90)
Educational background	Technical secondary school	1 (0.10)
	Diploma or associate degree	246 (17.00)
	Bachelor’s degree	1,187 (82.10)
	Master’s degree and above	12 (0.80)
Hospital classification	General hospital	1,331 (92.00)
	Specialized hospital	115 (8.00)
Professional rank	Primary	932 (64.50)
	Intermediate	485 (33.50)
	Senior	29 (2.00)
Clinical role	Nurse	1,313 (90.80)
	Head nurse	130 (9.00)
	Director of the nursing	3 (0.20)
Rescue experience (years)	0	852 (58.90)
	1–2	513 (35.50)
	3–4	51 (3.50)
	≥ 5	30 (2.10)
Clinical experience (years)	< 5	464 (32.10)
	5–10	470 (32.50)
	10–15	280 (19.40)
	15–20	142 (9.80)
	≥ 20	90 (6.20)

*N*: number; IQR: interquartile range.

**Figure 1:**
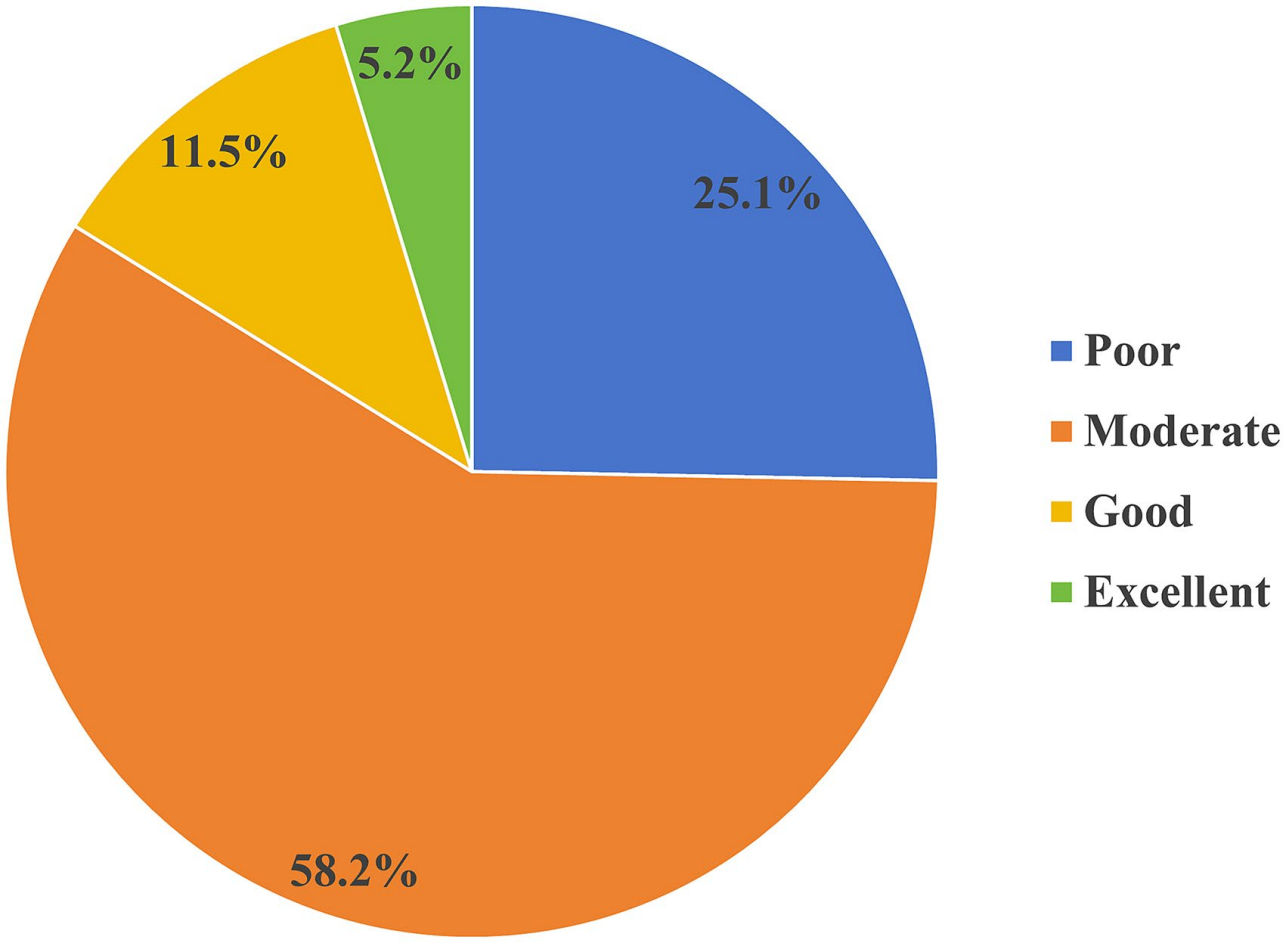
The level of nurses’ public health emergency response capacity for EIDs.

**Figure 2:**
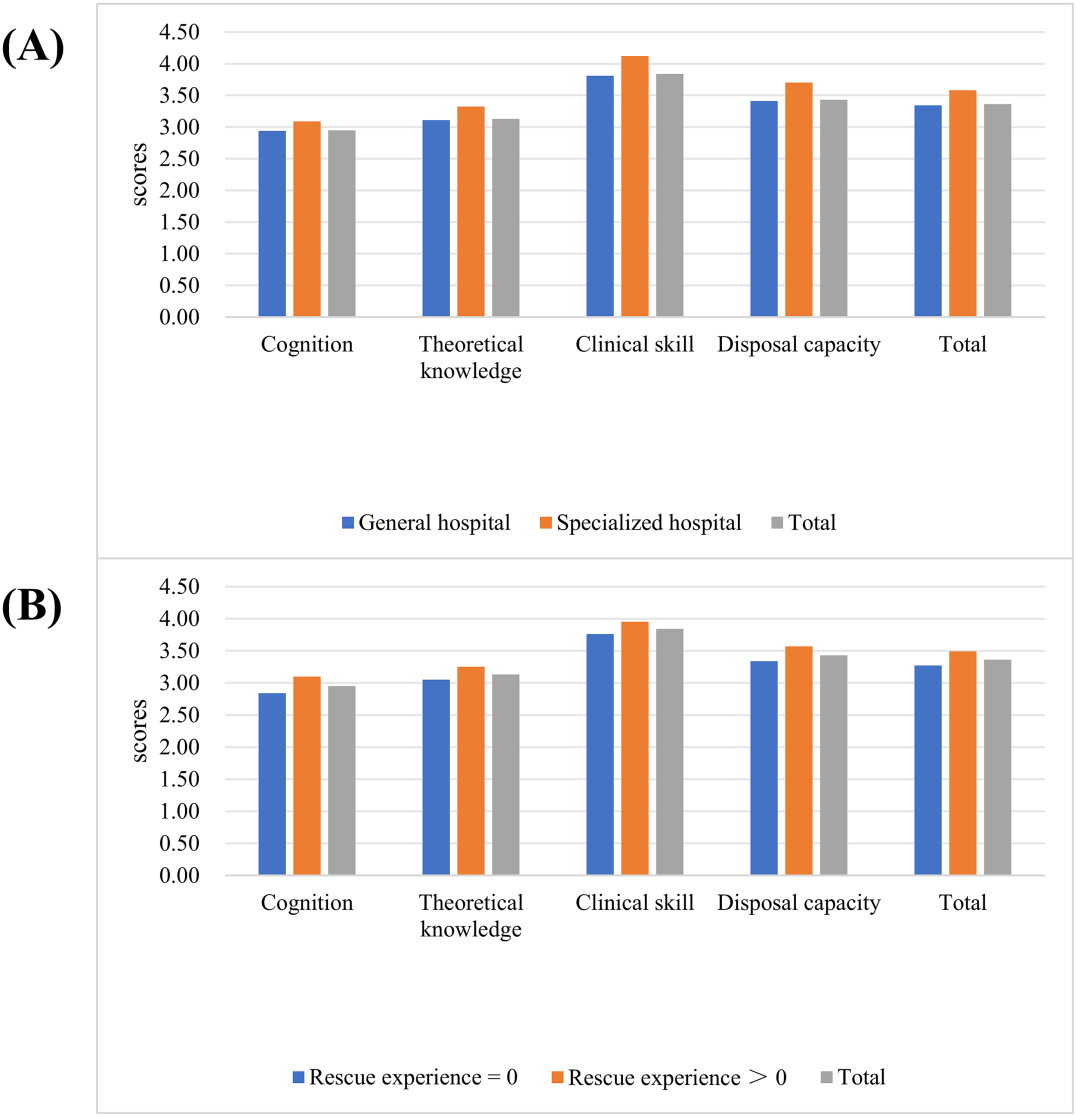
The scores of public health emergency response capacity for EIDs. **(A)** The scores of public health emergency response capacity for EIDs among nurses in different hospitals. **(B)** The scores of public health emergency response capacity for EIDs among nurses with different rescue experience frequency.

The scores of the nurses’ public health emergency response capacity for EIDs ranged from 54 to 270 (median = 177, IQR = 161–203). 25.3% of clinical nurses achieved scores below 60% of the total questionnaire, indicating a poor level of emergency response capacity, 58.5% of clinical nurses scored between 60 and 79%, reflecting a moderate level of emergency response capacity, 11.5% of clinical nurses scored between 80 and 89%, demonstrating a good level of emergency response capacity, and 4.7% attained scores of 90% and above, with an excellent level of emergency response capacity (see Figure 1). Furthermore, the analysis revealed statistically significant disparities in the scores of the dimensions of nurses’ emergency response competencies (H = 1146.228, *p* = 0.000). The scores of the dimensions were ranked in ascending order as follows: cognition for EIDs, theoretical knowledge for EIDs, disposal capacity for EIDs, and clinical skill for EIDs (see Figure 2).

The results of the analysis showed that significant differences were observed in the scores across the cognitive dimensions and theoretical knowledge dimensions of emergency response capability on the hospital classification and rescue experience (*p* < 0.05). The scores of the clinical skill dimension in emergency response capability exhibited significant differences across age (*p* = 0.013), gender (*p* = 0.008), professional rank (*p* = 0.000), clinical role (*p* = 0.000), hospital classification (*p* = 0.000), clinical experience (*p* = 0.000) and rescue experience (*p* = 0.000). Moreover, there was also a significant difference in the disposal capacity of emergency response capacity among nurses with different clinical roles (*p* = 0.049), hospital classification (*p* = 0.000) and rescue experience (*p* = 0.000). Besides, there was a significant difference in scores for nurses’ public health emergency response capacity for EIDs in terms of clinical role (*p* = 0.008), hospital classification (*p* = 0.000), and rescue experience (*p* = 0.000) (see Table 2 and Figure 2).

**Table 2:** Univariate analysis of the different characteristics and nurses’ emergency response capacity (*n* = 1,446).

Variable		Dimension of cognition Median (IQR)	Dimension of theoretical knowledge Median (IQR)	Dimension of clinical skill Median (IQR)	Dimension of disposal capacity Median (IQR)	Total Median (IQR)
Age		3.00 (2.50–3.20)	3.00 (2.75–3.46)	3.86 (3.29–4.36)	3.36 (2.93–4.00)	177.00 (161.00–203.00)
H		41.647	34.920	56.153	32.384	32.399
*P*		0.204	0.472	0.013*	0.595	0.594
Gender	Female	3.00 (2.50–3.20)	3.00 (2.75–3.50)	3.93 (3.29–4.36)	3.36 (2.93–4.00)	177.00 (161.00–203.00)
	Male	3.00 (2.90–3.10)	3.00 (3.00–3.32)	3.50 (3.00–4.14)	3.14 (3.00–4.00)	172.00 (162.00–197.50)
Z		3.078	0.887	7.058	0.417	0.070
*P*		0.079	0.346	0.008*	0.518	0.792
Educational background	Technical secondary school	1.10	1.13	2.36	1.00	76.00
	Diploma or associate degree	3.00 (2.50–3.30)	3.00 (2.75–3.50)	3.86 (3.00–4.36)	3.36 (3.00–4.00)	176.00 (162.00–205.00)
	Bachelor’s degree	3.00 (2.50–3.20)	3.00 (2.75–3.44)	3.86 (3.36–4.36)	3.36 (2.93–4.00)	177.00 (161.00–203.00)
	Master’s degree and above	2.95 (2.35–3.85)	3.06 (2.94–4.05)	4.11 (3.66–4.55)	3.64 (3.09–4.00)	188.50 (174.25–210.75)
H		4.064	4.356	6.682	5.753	4.799
*P*		0.255	0.255	0.083	0.124	0.187
Professional rank	Primary	3.00 (2.50–3.30)	3.00 (2.75–3.50)	3.86 (3.21–4.29)	3.36 (2.95–4.00)	176.00 (161.00–203.75)
	Intermediate	2.90 (2.40–3.20)	3.00 (2.75–3.44)	4.00 (3.43–4.43)	3.36 (2.93–3.93)	178.00 (162.00–202.50)
	Senior	3.00 (2.50–3.30)	3.00 (2.78–3.50)	4.14 (3.57–4.47)	3.50 (2.93–4.25)	181.00 (160.50–210.00)
H		4.551	0.260	19.090	0.329	1.582
*P*		0.103	0.878	0.000*	0.848	0.453
Clinical role	Nurse	3.00 (2.50–3.20)	3.00 (2.75–3.44)	3.86 (3.29–4.36)	3.36 (2.93–4.00)	175.00 (161.00–202.00)
	Head nurse	3.00 (2.50–3.30)	3.06 (2.88–3.56)	4.14 (3.64–4.57)	3.50 (3.05–4.07)	187.00 (165.00–208.00)
	Director of the nursing	3.30 (2.35–3.85)	3.50 (2.78–3.94)	4.21 (4.07–4.39)	3.50 (3.00–3.78)	197.00 (167.00–216.00)
H		0.637	5.301	23.512	6.047	9.776
*P*		0.727	0.071	0.000*	0.049*	0.008*
Hospital classification	General hospital	3.00 (2.50–3.20)	3.00 (2.75–3.38)	3.86 (3.29–4.36)	3.29 (2.93–4.00)	175.00 (161.00–201.00)
	Specialized hospital	3.00 (2.60–3.50)	3.19 (2.88–3.94)	4.21 (3.71–4.71)	3.79 (3.07–4.21)	193.00 (166.00–218.00)
Z		4.763	10.552	23.098	16.181	16.692
*P*		0.029*	0.001*	0.000*	0.000*	0.000*
Clinical experience (years)	< 5	3.00 (2.60–3.20)	3.00 (2.81–3.44)	3.71 (3.07–4.21)	3.29 (3.00–4.00)	174.00 (161.00–200.75)
	5–10	3.00 (2.40–3.20)	3.00 (2.69–3.50)	3.93 (3.29–4.36)	3.36 (2.93–4.00)	176.50 (159.00–203.25)
	11–15	2.90 (2.40–3.20)	3.00 (2.75–3.38)	4.00 (3.43–4.43)	3.36 (2.86–3.93)	178.00 (162.00–200.00)
	16–20	3.00 (2.60–3.40)	3.00 (2.81–3.63)	3.97 (3.50–4.50)	3.50 (3.00–4.00)	183.00 (161.75–209.00)
	≥ 20	3.00 (2.60–3.30)	3.00 (2.86–3.46)	3.97 (3.48–4.50)	3.40 (2.91–4.02)	179.00 (163.75–203.00)
H		8.841	6.710	27.701	2.090	4.703
*P*		0.065	0.152	0.000*	0.719	0.319
Rescue experience (times)	0	2.90 (2.30–3.10)	3.00 (2.69–3.30)	3.71 (3.21–4.29)	3.21 (2.86–3.86)	172.00 (158.00–196.00)
	1–2	3.00 (2.60–3.40)	3.00 (2.81–3.69)	4.00 (3.36–4.43)	3.50 (3.00–4.00)	180.00 (162.00–208.50)
	3–4	3.35(2.98–3.90)	3.31 (3.00–3.91)	4.11 (3.84–4.52)	3.83(3.41–4.31)	198.00 (179.75–221.00)
	≥ 5	3.10(2.60–3.70)	3.25 (3.00–3.94)	4.21 (3.93–4.64)	3.86(3.43–4.29)	201.00 (172.00–219.00)
H		57.122	38.170	38.041	49.083	58.325
*P*		0.000*	0.000*	0.000*	0.000*	0.000*

*N*: number; IQR: interquartile range; *significance as *p*-value < 0.05. The bold values indicate statistical significance (*P* < 0.05).

The findings showed that the hospital classification and rescue experience affected the nurses’ public health emergency response capacity for EIDs (R^2^ = 0.044, *F* = 11.023, *p* = 0.000 < 0.05). Nurses at specialized hospitals exhibited superior theoretical knowledge, clinical skill, and emergency disposal ability for EIDs compared to those in general hospitals (*p* < 0.05). Nurses who had more rescue experience demonstrated a higher level of cognitive and theoretical knowledge, clinical skill, and emergency disposal ability of EIDs (*p* < 0.05). Besides, the clinical role and clinical experience influenced the performance of nurses’ clinical skills in EIDs (*p* = 0.000 < 0.05). The nursing team leaders performed better in clinical skills. Nurses with 10–15 years of clinical experience exhibited superior clinical skill levels compared to nurses with fewer than 5 years of clinical experience (see Table 3).

**Table 3:** Multiple linear regression analysis of factors associated with emergency response capability scores (*n* = 1,446).

Variable		β	t	*P*	95% CI	R^2^	F
Cognitive level	Hospital classification (ref. general hospital)					0.037	11.753*
	Specialized hospital	0.025	0.941	0.347	(−0.073–0.207)		
	Rescue experience (times) (ref. 0)						
	1–2	0.155	5.874	0.000	(0.158–0.316)		
	3–4	0.083	3.133	0.002	(0.123–0.535)		
	≥ 5	0.106	4.068	0.000	(0.283–0.809)		
Theoretical knowledge	Hospital classification (ref. general hospital)					0.031	11.389*
	Specialized hospital	0.060	2.282	0.023	(0.020–0.270)		
	Rescue experience (times) (ref. 0)						
	1–2	0.125	4.717	0.000	(0.099–0.241)		
	3–4	0.082	3.076	0.002	(0.105–0.473)		
	≥ 5	0.083	3.159	0.002	(0.144–0.614)		
Clinical skill	Gender (ref. female)					0.055	6.451*
	Male	−0.050	−1.908	0.057	(−0.347–0.005)		
	Professional rank (ref. primary)						
	Intermediate	0.017	0.467	0.641	(−0.078–0.126)		
	Senior	−0.006	−0.201	0.840	(−0.325–0.264)		
	Clinical role (ref. nurse)						
	Head nurse	0.071	2.252	0.024	(0.021–0.309)		
	Director of the nursing	0.020	0.761	0.447	(−0.462–1.048)		
	Hospital classification (ref. general hospital)						
	Specialized hospital	0.091	3.459	0.001	(0.098–0.354)		
	Rescue experience (times) (ref. 0)						
	1–2	0.087	3.290	0.001	(0.049–0.195)		
	3–4	0.091	3.378	0.001	(0.139–0.525)		
	≥ 5	0.062	2.349	0.019	(0.048–0.531)		
	Clinical experience (years) (ref. <5)						
	5–10	0.054	1.747	0.081	(−0.009–0.164)		
	10–15	0.080	2.225	0.026	(0.016–0.255)		
	15–20	0.064	1.856	0.064	(−0.008–0.297)		
	≥ 20	0.013	0.375	0.708	(−0.155–0.228)		
Disposal capacity	Clinical role (ref. nurse)					0.037	9.308*
	Head nurse	0.015	0.552	0.581	(−0.096–0.171)		
	Director of the nursing	−0.015	−0.568	0.570	(−1.060–0.584)		
	Hospital classification (ref. general hospital)						
	Specialized hospital	0.072	2.728	0.006	(0.055–0.336)		
	Rescue experience (times) (ref. 0)						
	1–2	0.113	4.242	0.000	(0.093–0.254)		
	3–4	0.113	4.205	0.000	(0.240–0.660)		
	≥ 5	0.083	3.168	0.002	(0.163–0.694)		
Emergency response capacity	Clinical role (ref. nurse)					0.044	11.023*
	Head nurse	0.024	0.907	0.365	(−3.193–8.682)		
	Director of the nursing	0.002	0.068	0.946	(−35.375–37.928)		
	Hospital classification (ref. general hospital)						
	Specialized hospital	0.074	2.809	0.005	(2.711–15.264)		
	Rescue experience (times) (ref. 0)						
	1–2	0.135	5.093	0.000	(5.709–12.861)		
	3–4	0.105	3.927	0.000	(9.384–28.117)		
	≥ 5	0.095	3.628	0.000	(10.048–33.710)		

β: the standardized regression coefficient; CI: confidence interval; *: significance as *p*-value < 0.05; ref: reference category. The bold values indicate statistical significance (*p* < 0.05).

## 4 Discussion

This cross-sectional investigation of 1,446 registered nurses from tertiary hospitals in Beijing revealed multidimensional characteristics of public health emergency response capacity for EIDs. The results showed that nurses’ clinical skill scores (median = 3.86, IQR = 3.29–4.36) significantly surpassed the scores of theoretical knowledge (median = 3.00, IQR = 2.75–3.46) and cognitive abilities (median = 3.00, IQR = 2.50–3.20), while disposal capacity (median = 3.36, IQR = 2.93–4.00) demonstrating intermediate performance. The majority of nurses’ existing emergency response capacity featured poor cognitive status, inadequate theoretical knowledge, suboptimal disposal capacity, yet proficient clinical skills. This may impede their ability to do in-depth analysis for EIDs. This result corroborated the findings reported by He et al. (11) and Gu and Wang (24). This competence imbalance may be ascribed to the repeated hands-on practices in clinical settings that have strengthened skill proficiency, while the theoretical knowledge of emergency response for non-routine applications was partially forgotten after the pandemic. However, previous research has suggested that nurses’ specialized emergency response knowledge is essential for achieving high-quality emergency services (25). Alfuqaha’s et al. (26) study also pointed out that knowledge and skills are intricately connected and knowledge is the basis of skills. Moreover, knowledge affects action (disposal capacity) via the intermediary function of cognition or attitude (27). Consequently, we propose an intervention strategy focusing on theoretical reinforcement to foster positive cognition and attitudes via systematic knowledge building, which in turn enhances disposal capacity and ultimately enabling the synergistic development of competence in all dimensions.

Additionally, the median score for nurses’ emergency competence was 177, which is at the level of 65.5%. It was also found that 25.3% of clinical nurses exhibited a poor level of emergency response capacity, 58.5% showed a moderate level of emergency response capacity, and only 16.2% had a good or excellent level of emergency response capacity. The overall emergency response capacity of nurses was suboptimal. The findings aligned with the conclusions drawn in prior investigations (16, 28, 29). The possible reasons need to be analyzed at a systemic level. Firstly, due to the sudden and minimal epidemiological occurrence of EIDs (9, 30), nurses, especially those in general departments, have limited opportunities to participate in public health crises. Nurses lack experience and opportunities to develop their expertise (11). However, in our study, we included nurses from both related and unrelated departments. So, the scores of nurses’ emergency competence were not high. Secondly, this may relate to the inherent complexity of EIDs. Emergency response capacity for EIDs is a comprehensive capability encompassing the whole disaster process and all-hazards, which not only requires nurses to have specialized knowledge, but also needs to have a strong adaptive capacity (8). Moreover, it is also influenced by the working environment, medical equipment and other factors. Therefore, executing measures to enhance nurses’ emergency response capacity constitutes an urgent imperative. Recent researchers have reported that adequate scientific education and training are important for improving nurses’ emergency response capabilities (31, 32), as they not only promote knowledge acquisition but also transform nurses’ cognitive-behavioral adaptation patterns, strengthen nurses’ capacity and confidence in responding to EIDs. Although emergency training has been implemented, nurses’ emergency response proficiency remains suboptimal (33). This may be associated with current paradigms in education and training. In China, formal higher education and structured clinical continuing education programs form the primary pillars of nurse education (34). However, infectious disease-related content remains limited in higher education courses (35). Furthermore, the course on infectious disease nursing has not been established as a distinct discipline. So, the current curricular content is deficient in specialization. Meanwhile, in clinical continuing education, there is an excessive emphasis on the knowledge within the department and perhaps neglect the development of infectious disease specialty competencies (36). Therefore, in accordance with WHO’s sustained capacity-building framework, we recommend that it is necessary to systematically integrate lessons learned from previous infectious disease outbreaks to design specialty courses (20). Besides, it is essential to establish a dynamic updating mechanism to regularly update and adapt educational and training programs based on the reality of EIDs (37, 38). To ensure the continuous development of nurses’ emergency response capacity, managers can adopt a phased and intensive training model that integrates both online and offline components, while also creating high-quality online training courses and improving the online training system to address unforeseen pandemic scenarios (33).

Notably, previous studies have shown that nurses’ willingness and mindset to cope with EIDs negatively predicted nurses’ emergency response capacity (39). Managers should incorporate psychological resilience development into emergency training protocols, enhancing nurses’ psychological adaptive capacity to facilitate their effective engagement in response efforts during EIDs. Concurrently, it is also important to pay attention to the continuous evaluation of emergency response capability after training (16), and if necessary, it can be combined with artificial intelligence (AI) to conduct personalized analysis of nurses’ emergency response capability and create a personalized learning pathway. This will enable the emergency response training model of training-assessment-feedback to realize a closed loop. More importantly, there exists a deficiency in the cultivation of expert nurses in infectious diseases. However, Gorjian et al. (35) emphasized that specialized nurses with specific qualifications can provide evidence-based support for emergency decision-making and effectively respond to outbreaks of infectious disease. So, the construction of specialized nurse system in EIDs will significantly enhance the overall capacity improvement.

Besides, our study has identified that rescue experience in EIDs was a critical predictor of nurses’ emergency response capacity (20), which significantly impacted the performance in all competency dimensions. Previous researchers have also shown the importance of rescue experience in improving emergency response capabilities (20, 34, 40). A qualitative investigation (41) revealed that nurses with rescue experience displayed higher initiative in enhancing domain-specific knowledge and skills, exhibited stronger response proficiency, and formed greater capacity to respond psychological impacts of EIDs. Therefore, we believe that emergency simulations may be a high-effective method for assessing and improving nurses’ emergency response proficiency (39). And its widespread implementation deserves to be seriously considered by managers. However, our investigation failed to demonstrate statistically significant differences between the emergency response capacity and the frequency of rescue experience. This finding probably reflects critical deficiencies in post-crisis evaluation processes. Additional examination of these processes is necessary.

In our study, significant differences were observed in the dimension of clinical skill and disposal capacity, as well as the overall level of emergency response competence between nurses and nurse managers. Nurse leaders were doing better than nurses. Park et al. (42) also pointed out that higher positions, the better the emergency response skills of the nurses. Nevertheless, due to the limited sampling of nurses above the position of head nurse included in our research, we could not identify a correlation between the emergency response capacity of nurses above the position of head nurses and head nurses. Notably, no significant differences in cognition and theoretical knowledge about EID between nurses and head nurses were reported in our study. Some studies have even shown that some nursing educators and administrators themselves demonstrate suboptimal proficiency in disaster management (43). Benner’s novice to expert model (44, 45) posits that the progression of competence relies on the accumulation of contextualized practice experience rather than mere knowledge accumulation. Although nurse managers often have richer practical experience, it remains undeniable that they also need to enhance theoretical knowledge and awareness of EIDs. Neglecting to these gaps will undermine their guidance effectiveness and reduce their professional reputation among staff nurses. Therefore, we suggest that hospital management prioritize the training and education of nurse leaders to maximize their guiding role and positively guide ordinary nurses. Furthermore, due to the differences in the emergency competency and the training requirements of clinical nurses, we propose formulating differentiated emergency response training programs based on the nurses’ characteristics of various clinical roles.

Meanwhile, we also found that the categorization of hospitals impacted nurses’ emergency capacity. Nurses at specialized hospitals demonstrated superior overall emergency response capabilities and all dimensions of emergency response capabilities. Wang’s research (36) indicated that specialized infectious disease hospitals exhibited higher occupational exposure risks than general hospitals. So, nurses at specialized institutions demonstrated higher preventive awareness and more comprehensive understanding of EIDs. Furthermore, nurses at specialized infectious disease hospitals get substantially more opportunities to participate in major epidemic control efforts. Their accumulated frontline experience in crisis management contributes to enhancing professional confidence when confronting public health emergencies, demonstrating stronger self-efficacy in addressing pandemic-scale infectious disease challenges. Finally, these observed differences may be associated with the differences in organizational settings and organizational culture (46). However, within China’s healthcare system, general hospitals undertake response operations of a greater scale and frequency. Therefore, we recommend intensifying emergency response training and simulations for nurses in general hospitals to reduce performance gaps compared to specialized infectious disease institutions. Notably, the WHO Strategic Framework for Emergency Preparedness advocates for a whole-of-government, whole-of-society approach. So, we suggest the establishment of cross-organizational coordination between specialized and general hospitals to enhance the emergency response capacity of the whole nursing workforce.

Last but not least, nurses exhibit inadequate cognitive awareness of EIDs. Nurses in general departments may think that infectious disease outbreaks are more relevant to specialized areas such as infectious diseases and emergency departments. Discrepancies in role perception may further intensify the competency gap (36). Hong’s research also indicated that nurses’ willingness to respond to disasters is critical to disaster capacity. Besides, Michie’s behavior system: capability, opportunity, and motivation model (47) argued that the efforts to build emergency response capacity just through training are insufficient. To meet the health workforce criteria specified in the WHO Strategic Framework for Emergency Preparedness and to achieve sustainable development of emergency response capacity, it requires capability (knowledge training or skill training), opportunity (defined role duties and healthcare resources) and motivation (leadership by example, support from leaders or organizations and remuneration systems) to synchronously develop. When the organizational opportunity barriers are removed, knowledge construction and motivational activation will generate synergistic effects, ultimately realizing system-wide enhancement of emergency response capabilities. Hence, it is essential to clarify nurses’ awareness of their roles and responsibilities in public health outbreaks of EIDs (48), which motivates nurses to provide the safest, highest-quality, and most prudent care in emergencies (49).

## 5 Limitations

There are some limitations in this study. Firstly, this study was conducted only in 3 tertiary hospitals in Beijing. Furthermore, the use of a convenience sampling method may induce potential selection bias and limit the representativeness of the research participants. Consequently, the generalizability of the results is constrained. In the future, we ought to use stratified random sampling techniques in various healthcare settings. Secondly, as a cross-sectional study, this study cannot infer directional or causal relationships, just observed associations. Controlled trials will be needed in the future to draw further conclusions and explore the mechanisms of their effects. Finally, due to the significant gender discrepancy and uneven hospital-type representation, the study’s findings should be interpreted and applied to other study conditions with caution.

## 6 Conclusion

The multicenter investigation of nurses at Beijing’s tertiary hospitals revealed a modest emergency response capacity for EIDs with significant inter-dimensional imbalances. Inadequate knowledge mastery and cognitive attitudes about EIDs impair nurses’ behavioral performance during emergency responses. A critical finding from this study revealed that the hospital classification and the rescue experience of EIDs impacted nurses’ emergency response capabilities. Especially, the rescue experience showed significant predictive capability. To enhance nurses’ emergency response capabilities to respond to EIDs, we recommend consolidating theoretical reinforcement training and cognitive transformation programs for nurses. Employing emergency simulations as the high-efficacy pathways for evaluating and improving nurses’ emergency response capacity. Simultaneously, accelerating the cultivation of specialized nurses in EID and implementing cross-institutional collaboration is essential. Driven by educational reform, organizational incentives, and leadership modeling, this integrated approach may foster a WHO-compliant nursing workforce equipped with resilient and sustainable emergency response capacities. Finally, the conclusions we drew were solely based on our study population. Numerous and various types of future studies are needed to explore the more influential factors of nurses’ emergency response competence and to enhance the improvement of nurses’ emergency response capacity for EIDs.

## Data Availability

The raw data supporting the conclusions of this article will be made available by the authors, without undue reservation.
